# Endoscopic plication for the treatment of twisted pouch

**DOI:** 10.1093/gastro/goae065

**Published:** 2024-06-27

**Authors:** Bo Shen

**Affiliations:** Center for Inflammatory Bowel Disease and the Global Center for Integrated Colorectal Surgery and IBD Interventional Endoscopy, Columbia University Irving Medical Center/New York Presbyterian Hospital, New York, NY, USA

## Introduction

Restorative proctocolectomy with ileal pouch–anal anastomosis (IPAA) is a preferred surgical procedure for patients after colectomy for medically refractory ulcerative colitis (UC), -associated colitis, or familial adenomatous polyposis. While the stoma-avoiding procedure improves patients’ health-related quality of life, structural and inflammatory adverse sequelae are common. Floppy pouch complex is one of the common structural complications of ileal pouch [1]. Twisted pouch or pouch volvulus is a clinical phenotype of floppy pouch complex [2, 3]. Pouchoscopy and pouchogram are the main modalities for the diagnosis [4, 5]. Twisted pouch has traditionally been treated with surgical de-rotation, pexy, pouch redo, or excision [2, 6–8]. Patients are at risk of postoperative recurrence or pouch excision [4, 5]. Less invasive, endoscopic approaches have been performed for the treatment of twisted pouches such as septectomy [9]. Here we described the first case in the literature in which symptomatic twisted pouch was successfully treated with endoscopic plication.

## Case report

An 18-year-old female presented with frequent bowel movements, bloating, urgency, incomplete evacuation, fatigue, and weight loss at our Pouch Clinic. She was diagnosed with UC at age 11 years and had two-stage IPAA at age 14 years for refractory UC with a history of *Clostridium difficile* infection. Since surgery, she had never felt right and gradually developed the presenting symptoms. She had been treated with long-term oral ciprofloxacin or metronidazole for “pouchitis” or hydrocortisone suppositories for cuffitis with minimal improvement in symptoms. Cross-sectional imaging at a local facility showed a dilated pouch and pre-pouch ileum. Past medical history showed mild anxiety and depression, and no extra-intestinal manifestation of inflammatory bowel disease.

**Figure 1.**
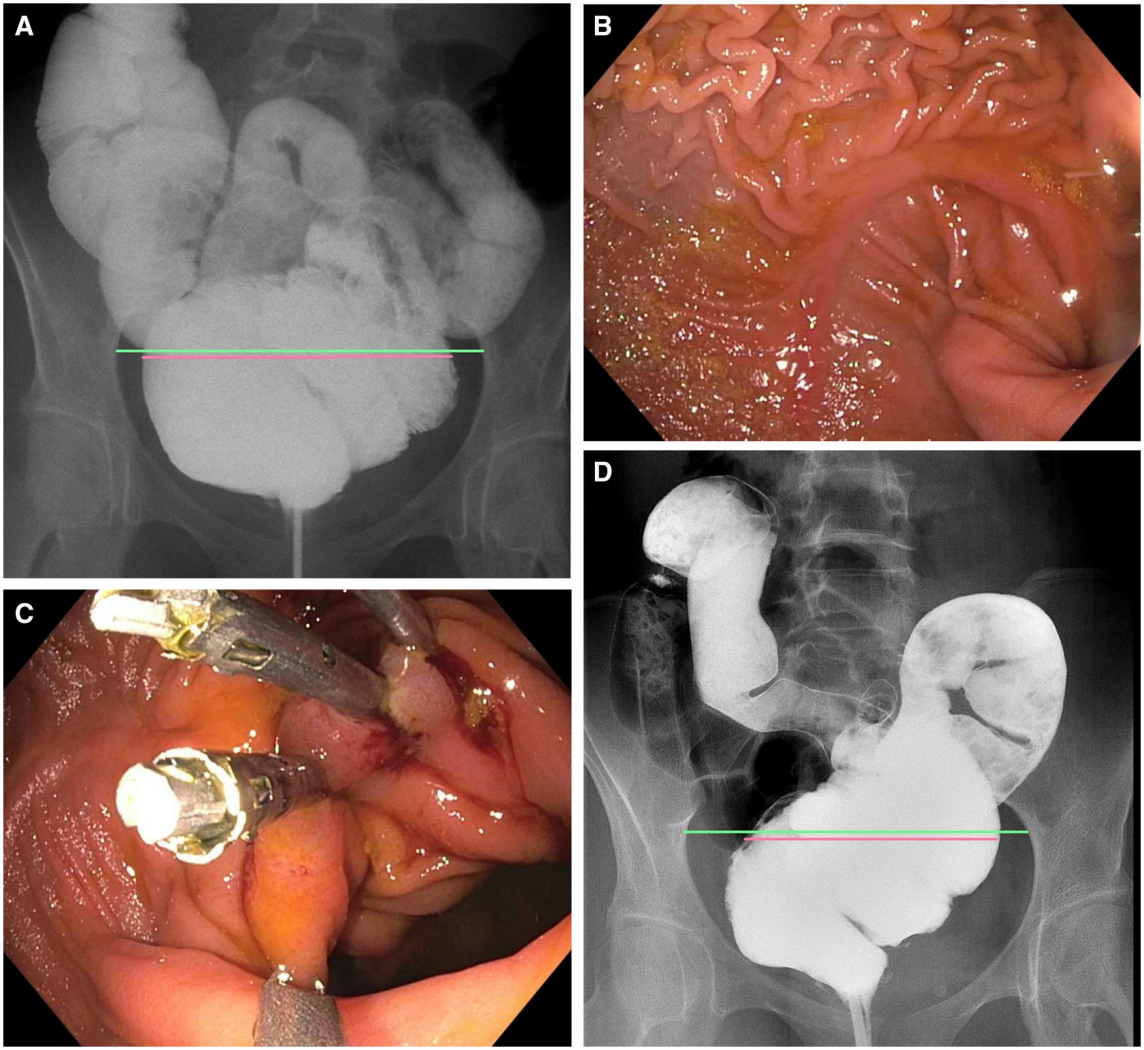
Twisted pouch before and after endoscopic plication. (A) Twisted pouch body with dilated pouch body and pre-pouch ileum before therapy. Notice the ratio of the pouch body (pink line) and the pelvic inlet ring (green line). (B) Twisted pouch body on endoscopy before therapy. (C) Placement of endoclips for the plication of pouch twist. (D) The twisted pouch post endoscopic therapy. Notice the shrunken size of the pouch body with a reduced ratio between the pouch body (pink line) and the pelvic inlet ring (green line), along with the reduced size of the lumen of the pre-pouch ileum.

At the Pouch Center at Columbia University, a physical examination showed a distended abdomen that was tympanic to percussion. Barium defecography showed a slow-emptying, twisted, and dilated pouch body (with a pouch body/pelvic inlet ratio of 7.31) and a dilated pouch ileum (Figure 1A). Diagnostic pouchoscopy showed normal perianal and digital rectal examinations showed long pouch body with normal mucosa but axial twists in the mid-pouch body (Figure 1B), angulation at the pouch inlet, and dilated lumen of the pre-pouch ileum. There were no signs of ischemia or necrosis. Histology of segmental biopsie showed non-specific inflammation at the pre-pouch ileum, pouch body, and cuff. Anopouch manometry showed normal pouchanal inhibitory reflex but failed balloon expulsion and the presence of pelvic dyssynergia. She was diagnosed with structural and functional pouch outlet obstruction. She first underwent 10 sessions of biofeedback therapy without improvement in symptoms.

A subsequent outpatient therapeutic pouchoscopy was performed. The anatomy (i.e. twisted pouch) and mucosal pattern of the pre-pouch ileum, pouch body, and cuff were similar to the diagnostic pouchoscopy 4 months prior. Under monitored anesthesia care, the patient’s twisted pouch was treated with endoscopic plication with eight endoclips (MANTIS^®^ clip, Boston Scientific, Marlborough, MA, USA) to partially untwist the pouch body (Figure 1C). The patient tolerated the procedure well and was discharged home after a 45-minute observation at the post-procedure care unit. She reported improved urgency and dyschezia, and reduced stool frequency 2 weeks after the endoscopic intervention.

Repeat barium defecography 6 months later showed improved dilation of the pouch (with a pouch body/pelvic inlet ratio of 7.13) (Figure 1D), cuff, and pre-pouch ileum. Repeat pouchoscopy showed that the previously dilated pouch body and pre-pouch ileum, as well as the twisted pouch body, had improved. Since the patient had had a favorable response in symptoms, imaging, and pouchoscopy to the therapy, her twisted pouch was further treated with endoscopic plication 7 months later. The patient was subsequently scheduled to have a yearly pouchoscopy for disease monitoring, dysplasia surveillance, and endoscopic plication of the twisted pouch as needed.

## Discussion and conclusions

With unclear etiopathogenesis, pouch volvulus or axial pouch twist is a rare complication of IPAA. The estimated frequency is 0.18% (3/1,700) [5]. While surgery has been considered standard care, endoscopic decompression and detortion are used for temporary symptom relief and provide a bridge for subsequent therapy [5]. In patients with partial pouch twist and absence of ischemia or necrosis, more definitive endoscopic therapy can be attempted. For patients with distal pouch twists, endoscopic septectomy with an insulated knife or needle knife has been reported [6]. Untreated pouch twist can lead to ischemia or necrosis of the bowel at the tip and further dilation of proximal bowel, and eventual pouch failure.

Pouch twist is considered a phenotype of floppy pouch complex. Floppy pouch complex is common after IPAA for both UC and familial adenomatous polyposis. It is noted that its frequency may be related to the wide application of laparoscopic surgery with reduced adhesions to support the pouch or technical difficulties in visualization, mobilization, or construction of the bowel anastomosis. The main phenotypes of floppy pouch complex are pouch prolapse, pouchocele, afferent limb syndrome, efferent limb syndrome, axial twist (i.e. volvulus), and horizontal twist (i.e. pouch folding). As in this patient, floppy pouch complex often coexists with pelvic dyssynergia and mental disorders. Before endoscopic or surgical therapy, we recommend biofeedback therapy and treatment for anxiety or depression. The MANTIS^®^clip with its unique anchor prongs was initially designed for closure of large defects of the gastro-intestinal tract. The clip is wide and strong enough to hold pouch folds together in our endoscopic plication procedure.

Endoscopic management of inflammatory bowel disease and its postoperative complications have been explored and performed [10]. The endoscopic treatment modalities include balloon dilation of stricture, stricturotomy, strictureplasty, sinusotomy, fistulotomy, septectomy, banding ligation (pouch prolapse and pouchocele), and resection (colitis-associated neoplasia). This is the first description of endoscopic plication therapy for pouch twists or volvulus. Close monitoring and periodic endoscopic therapy are required for the patients.
